# Aspects of Multicellularity in *Saccharomyces cerevisiae* Yeast: A Review of Evolutionary and Physiological Mechanisms

**DOI:** 10.3390/genes11060690

**Published:** 2020-06-24

**Authors:** Monika Opalek, Dominika Wloch-Salamon

**Affiliations:** Institute of Environmental Sciences, Faculty of Biology, Jagiellonian University, Gronostajowa 7, 30-387 Kraków, Poland; monika.opalek@doctoral.uj.edu.pl

**Keywords:** facultative multicellularity, cell differentiation, cell specialization, cooperation, starvation, spatial structure, aging, metabolic cooperation, adaptation, evolution, *AMN1* gene, yeast exometabolome

## Abstract

The evolutionary transition from single-celled to multicellular growth is a classic and intriguing problem in biology. *Saccharomyces cerevisiae* is a useful model to study questions regarding cell aggregation, heterogeneity and cooperation. In this review, we discuss scenarios of group formation and how this promotes facultative multicellularity in *S. cerevisiae*. We first describe proximate mechanisms leading to aggregation. These mechanisms include staying together and coming together, and can lead to group heterogeneity. Heterogeneity is promoted by nutrient limitation, structured environments and aging. We then characterize the evolutionary benefits and costs of facultative multicellularity in yeast. We summarize current knowledge and focus on the newest state-of-the-art discoveries that will fuel future research programmes aiming to understand facultative microbial multicellularity.

## 1. Introduction

Evolutionary transitions from self-sufficient single cells to a multicellular organism are one of the most significant innovations in the history of life [1]. These and other changes include transitions from self-replicating genes to chromosomes and the evolution of free-living bacteria into eukaryote organelles, resulting in cooperation among previously independent smaller entities. The evolution of multicellularity enabled not only increased organismal complexity, but also adaptation to new environments.

Multicellularity evolved multiple times independently in various organisms′ lineages, including fungi [2,3]. Several multicellular forms and organismal complexity levels have been identified in the evolutionary tree of life. Complex, obligatory multicellularity comprises organisms with three-dimensional tissue organization and a genetically predetermined developmental program [4]. Complex multicellularity is known only in animals, plants, fungi, as well as brown and red algae [3]. For some lineages, however, multicellularity evolved without obligatory cellular differentiation. These simple, facultative multicellular organisms are able to switch between uni- and multicellularity in the course of their lives, and maintain this reversible state of cells in response to environmental conditions. Examples of such organisms can be found among Eukaryotes, Bacteria and Archaea [3].

**Figure 1:**
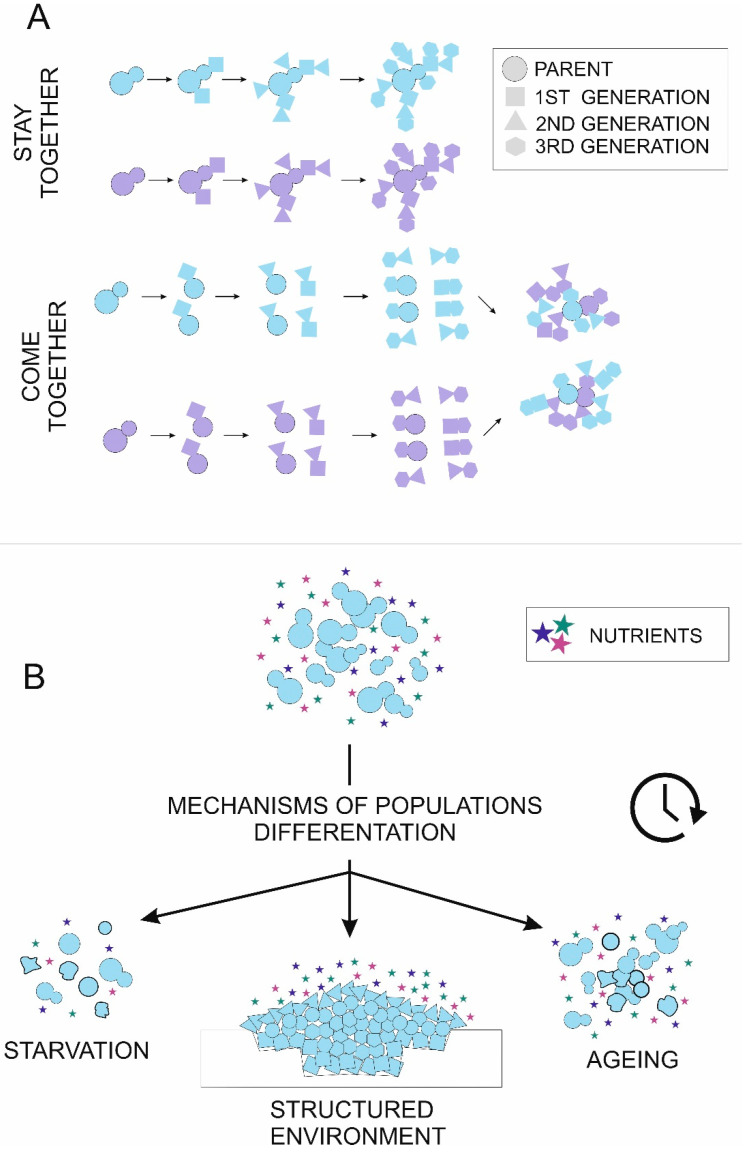
Mechanisms of cells aggregation (**A**) and cell differentiation (**B**) in multicellular yeast populations. (**A**) Colors symbolize cells′ genetic background. Cell shape reflects successive divisions. Cell aggregates can form by the “stay together” strategy, where daughter cells stay attached to the mother cell, leading to the formation of clonal adhesions. Alternatively, cells separate after each division and can aggregate later by cell wall adhesins (“come together”), leading to the formation of groups with random relations between cells. Both stay together and come together strategies lead to the formation of two aggregates with 16 cells each, but relatedness between adjacent cells differs. (**B**) There are three major environmental signals causing cell heterogeneity—nutrient limitation (starvation), structured environment and population aging. Initially, identical cells in the population can enter the apoptosis pathway (**uneven shape**), enter the quiescence pathway (**bold outline**) or specialize to perform various tasks (**cell shape**).

There are two steps in the evolution of facultative, simple multicellularity. First, cells form a group. Second, groups of cells differentiate, becoming heterogenous, then perform cooperative behaviour. Cell group forming can result from “staying together”, when a progeny cell remains attached to its ancestral cell, or “coming together”, when cells adhere to each other (Figure 1). Both scenarios are developed by *Saccharomyces cerevisiae*, a yeast which forms various multicellular structures: colonies, biofilms, mats, stalks, aggregates, flors and flocs (see Glossary) [5].

Cells growing as unicellular planktonic populations randomly interact with each other. However, cells within groups experience non-random interactions with neighbouring cells. The differentiation of individual cells within a group provides an interaction environment and promotes the evolution of cooperation (see Glossary), leading to adaptations beneficial to all engaged [6]. If cooperators interact preferentially with other cooperating cells, costs of cooperation (for example, production of public good—see Glossary) are outweighed by benefits (i.e., significantly higher availability of the public good, not shared with non-cooperating individuals) [7,8]. This preferentially creates conditions for the evolution of division of labour strategies (see Glossary). Task-specialized groups of cells may act more effectively, additionally reducing the costs of task-switching, which may be high for an individual cell [9,10]. A group of specialized cells can perform tasks with a level of efficiency that is impossible for a similar number of unicellular individuals [1,6]. As a result, a cooperating multicellular population acts as and experiences selection as a single organism entity.

In this review, we focus on the facultative multicellular yeast *Saccharomyces cerevisiae* as a model for studying the evolution of cell aggregation, differentiation and subsequent cooperation. We describe the tools that other researchers have used to investigate multicellularity in yeast. These include laboratory and molecular techniques, including genetic engineering and experimental evolution techniques used nowadays. Furthermore, we present recent exciting scientific advances based on metabolomics and community interactions as potentially fruitful future directions of research. We describe both the proximate mechanisms leading to heterogeneity in yeast and the entailing evolutionary benefits and costs.

## 2. Mechanisms and Methods to Study *Saccharomyces cerevisiae* Adhesion

*S. cerevisiae* cells grow vegetatively by budding. Each mother cell produces a daughter cell that may stay attached to the mother cell. This attachment between mother and daughter cells results in self-adhesion [11] and promotes a stay together strategy. There is a phenotypic variation regarding the self-adhesion trait among yeast strains. The majority of wild isolates form robust multicellular aggregates, the “clumpy phenotype”, and only few naturally occurring strains show a single-celled, planktonic life style [11]. Researchers have historically used a non-representative planktonic haploid *S. cerevisiae* for laboratory work, even though most wild *S. cerevisiae* strains are clumpy polyploids. The deliberate choice of non-adhesive planktonic type strains as laboratory models for worldwide usage, such as strains s288c and W303, has substantial consequences in the way *S. cerevisiae* has been perceived. A perspective that the planktonic type of growth is typical for yeast influenced knowledge about this important model organism biology for years. For example, using a planktonic type of strain (similar in this respect to bacteria), allowed the usage of many basic and indispensable microbiology laboratory techniques, such as the assessment of cell number based on optical density (OD) measurements and the determination of colony forming units (CFU) on agar media (e.g., for viability measurements). Nowadays it is clear to most yeast researchers that the presence of groups of cells formed by the strain has to be taken into consideration while using such methods.

Existing natural phenotypic variation in self-adhesion was first explained by genetic variation in the *AMN1* gene modulator of cell separation and mitotic exit [12]. A single missense substitution (an A to T change at nucleotide position 1104) resulted in an amino acid change (aspartic acid → valine) [12]. Most wild isolates have an aspartic acid codon in the genome and display an adhesive phenotype [13]. When the *AMN1* allele from a planktonic laboratory yeast (*S. cerevisiae* strain W303), was replaced using the classic genetic engineering method of homological recombination (see Glossary) with an allele from the adhesive wild yeast strain RM11, the laboratory strain acquired the adhesion phenotype of the wild yeast strain [14]. Analogously, the non-adhesive phenotype was found after introduction of the laboratory *AMN1* variant into genomes of wild strains [13].

In addition to genetic engineering, the stay together strategy as a mechanism for multicellularity was explored using experimental evolution methods. Experimental evolution research is extensively used to study adaptations and test evolutionary hypotheses in controlled laboratory conditions [15,16]. These experiments are designed in such a way that a given phenotype of interest will be favored during selection over generations. In the experimental evolution approach, there are no expectations for changes in particular genes made *a priori*, but differences in genomes of evolved and ancestral clones may be later analyzed to identify causative mutations.

The transcription regulator factor *ACE2* is involved in multicellularity repeatedly across experimentally evolved yeast populations. In the experimental evolution of multicellularity, gravity is used to select the sedimented bottom fraction of the population as the inoculum to start the next generation. In this way, bigger cell groups which sediment faster are promoted. After 60 transfers, primitive multicellularity, so-called snowflake forms, evolve [17]. Gene expression analysis allows for identification of ten downregulated genes. Seven of them: *CTS1*, *DSE4*, *DSE2*, *SUN4*, *DSE1*, *SCW11*, including already mentioned earlier, *AMN1*, are regulated by *ACE2*. Genetically engineered disruption of *ACE2* prevents daughter–mother cell separation and can re-create the snowflake phenotype [18,19]. Experimental evolution of the snowflake form was recently successfully applied to industrial beer strains [20]. Analogous reverse experimental evolution of unicellularity, where only the top fraction from the clumpy strain was transferred to the next generation, confirmed the causative role of the *AMN1* gene [21]. 

Cell–cell and cell–substrate adhesion promotes group formation by a come together strategy. In *S. cerevisiae* it is provided by the family of proteins that are anchored to the cell membrane and protrude from the cell wall, called flocculins [22]. They comprise seven different functional *FLO* genes, coding for five proteins involved in multicellularity and two proteins specific for conjugation of haploid cells in mating [23]. The role of flocculins in *S. cerevisiae* group formation has recently been specifically reviewed, so there is no need for us to go into the details [24]. The type of flocculins expressed determines the way yeast multicellular structures are formed. Flo11 proteins more specifically adhere to the same cell type, providing a stay together strategy. Other flocculins are non-specific, so could be used in both come together and stay together strategies. Specific cell–cell adhesion is conferred by the Flo11p protein, which binds to other Flo11p proteins. The strength of Flo11p binding is the highest for clonal cells and, as such, it can also serve the stay together strategy model [25]. The Flo11p has been also shown to be an essential flocculin for biofilm formation [26,27]. Proteins Flo5p, Flo9p and Flo10p allow for non-specific cell–cell and cell–substrate adhesion. Flo1p preferentially binds to other Flo1p proteins, but can bind non-specifically as well [28,29]. Performed experimental evolution of multicellularity within chemostat also confirmed the role of previously described transcription factor *ACE2* and the additional influence of flocculins, mainly the *FLO1* gene [30]. Such non-specific adhesion was used as a model for research on the evolution of the come together strategy [31]. Therefore, expression of flocculins in *S. cerevisiae* produces two distinct ways of sticking together and forming multicellular groups.

Both methodological approaches described here—genetic engineering and experimental evolution—indicated the prevalence of one mechanism of self-adhesion in yeast, dependent on cell wall structure between yeast mother and daughter cells. Chemostat wall growth conditions confirmed *FLO1* involvement in cell–substrate adhesion [30]. All these results show that such a small modification can dramatically change a strain′s phenotype from uni- to multicellular and have profound macroevolutionary consequences. Using experimental evolution and simple genetic engineering techniques, researchers are able to obtain collections of isogenic strains, different in one single nucleotide variation, which can be used for experiments testing hypotheses regarding the evolution of multicellularity. 

## 3. Mechanisms and Methods to Study *Saccharomyces cerevisiae* Cell Differentiation

The observation that vegetative cells of *S. cerevisiae* can adopt a multicellular growth form was made more than 100 years ago by Emil Hansen, who described the formation of large yeast aggregates (“skins”) in industrial strains after fermentation. Since then there have been several forms of growth described and defined in detail [11,32]. However, the three main types that we will focus on here are: planktonic growth, non-adhesive colony and adhesive biofilm. In a liquid medium, non-adhesive yeast cells are planktonic and will produce turbid cultures of individual cells, that might, however, form small aggregates of multiple cells [11]. Yeast forms so called “non-adhesive colonies” on solid agar media exposed to air. In order to develop non-removable biofilms, flocculins must be produced that confer foreign adhesion. Typically, cells in biofilms adhere to each other and to the foreign solid or semi-solid surface.

Cell differentiation of an initially identical, undifferentiated unicellular population is driven by the fitness advantages of heterogeneity, which can promote the possibility of division of labour between cells [6,33]. As an adaptation to specific life conditions even simple, unicellular organisms collectively perform tasks that cannot be as efficiently accomplished solitarily. At first, non-differentiated, genetically identical cells that stay connected physically can exchange chemical signals and metabolites. Independent, spontaneous changes in the regulatory mechanism of each cell, experiencing slightly different external and internal cues in its own microenvironment, leads to population heterogeneity. Such progressive diversification might lead in the most extreme case to the coexistence of growing cells, non-growing cells and even dead cells [6,34,35].

Except from stochastic effects, there are three general drivers of clonal microbe metabolic heterogeneity: nutrient limitation causing starvation, structured environment and aging [36,37] (Figure 1). Below, we present how these factors influence differentiation in groups of *S. cerevisiae* cells. In the cases of yeast biofilms and colonies, heterogeneity-promoting factors are usually coupled, which makes it difficult to study their effects separately.

### 3.1. Structured Environment

The presence of structure in the environment greatly influences the metabolism of yeast cells. Naturally a yeast cell would divide and form various multicellular structures. Changing the stability of the environment by applying various agar concentrations in the laboratory media results in various forms obtained: colonies, biofilms and aggregates (see Glossary) [11]. Recent research demonstrates that each individual yeast cell adjusts to changes in its microenvironment. This creates multicellular assemblies that are spatially self-organized through long-range metabolic interactions governed by physical rules of diffusion and uptake. Such an approach sheds new light on the genes′ coordinated actions in individual cells, in a biologically relevant multicellular context that has impact on ecology, evolution, development and the emergence of multicellularity. Below, we present recent research according to the order of complication of the yeast–substrate structure analyzed; from a few immobilized cells, through a single layer colony, to the most complicated ones, biofilms and colonies.

Experimental data show that the presence of a structured environment is enough to activate different metabolic genes, reflecting adaptation to certain environment niches. Gene expression of well-fed, immobilized yeast cells were compared with the planktonic type of grow cells [38]. This experiment was designed to study only the effect of environment structure, uncoupled from other factors influencing heterogeneity: starvation and aging. Remarkably, it was found that surface immobilization of yeast cells upregulates genes of glycolysis and cell wall biogenesis, as well as accelerating ethanol production and inducing glycogen accumulation. Contrary to yeast in a planktonic state, the immobilized cells rapidly cease division and preserve viability for more than two weeks [38].

Temporal oscillations between the activity of cells in the middle and the periphery naturally develop in two-dimensional colonies. Immobilization of the single yeast cell is an artificial concept, requiring a special experimental set up, preventing the cells from multiplying [38]. Another type of non-natural, but very informative, spatial yeast organization is a monolayer community of cells. This low complexity is enough to obtain heterogeneity and cooperation among initially clonal cells. A recently developed microfluidic device allows the growth of thin, extended arrays of yeast cell monolayers that are perfused with nutrients from a single direction. Remarkably, these yeast monolayers—two-dimensions colonies—were enough for cell differentiation and self-organization of metabolic processes over space and time [39]. The interior of these colonies is nutrient-limited as a consequence of nutrient consumption of cells on the outside. However, cells on the outside are dependent on metabolites produced in the interior, and the supply of these metabolites ceases when the interior is starved. This dependence leads to temporal oscillations, where cells in the interior and in the periphery alternate in their metabolic activity. Notably, it was shown that a growing extended assembly of cells presents a robust, steady-state spatial structure, transitioning between fermentative (high glucose environment, fast growth, rapid glucose utilization) and respiratory (low glucose environment, slow growth, slow but efficient glucose utilization) [40,41] regimes, located close to and far from the nutrient source, respectively.

Cells′ phenotypes differ greatly, depending on their location in the highly organized biofilm structure. In this review we focus on biofilm exclusively formed by one species, *S. cerevisiae*. Flo11p has been shown to be an essential flocculin for the formation and maintenance of the internal structure of biofilms [37,42]. The Flo11p adhesin is required for meiotic differentiation in *S. cerevisiae* minicolonies grown on plastic surfaces [43]. Cells at the bottom of the colony elongate and form unicellular pseudohyphae, which are able to penetrate the surface and collect nutrients more effectively than planktonic cells. Inside the colony there is a cavity surrounded by cells, producing an extracellular polymeric matrix (ECM). The ECM is of a glycosidic nature [44] and has been shown to provide water and nutrient storage for dividing cells. Non-dividing stationary phase cells make up the biofilm’s external layer. Two-photon microscopy of sliced agarose-encapsulated three-dimensional yeast colonies was used to show that cells that reside in different positions in an environmental gradient express different sets of genes, mediated by gene regulatory mechanisms. Cells adjust metabolism gene expression to a cell′s local microenvironment in the structured colony [45,46].

Extracellular production of metabolites seems to be an intrinsic property of yeast. This is a recent switch in the research framework, thanks to the presence of quantitative metabolite data. Before that, it was widely assumed that co-growing prototrophic yeast cells produce amino acid and nucleotide metabolites predominantly for themselves and export them at insufficient quantities to support co-growing cells. However, recent research reports that yeast colonies maintain a rich exometabolome and that cells exploit this metabolic pool preferentially over their own biosynthesis. The necessary condition for metabolic diversification is intercellular interaction among cells in the groups, so that individuals sense changes in their environment caused by other cells′ metabolites [47,48,49].

This process was studied by means of synthetic colonies, “self-establishing communities” that were able to cooperatively exchange metabolites [50,51]. The experimental set up combines classic yeast genetics tools with new technology. The community starts with prototrophic cells that have some of their own chromosomal metabolic genes disabled, and instead have a copy of these genes on small circular DNA “mini-chromosomes” (called plasmids). The gene on the plasmid can compensate for the yeast having its own gene missing and allows the cell to still make the metabolic product it needs to survive. However, as a single cell divides to form a colony, cells randomly lose these plasmids, leaving some of the cells deficient for a particular metabolite. These cells can only survive if they use amino acids that are released to the environment (as exomeatobolome) by other cells. Such metabolite exchange has been shown to effectively generate a very heterogeneous colony. The ultra-sensitive mass spectrometry method was used for detailed analysis of the intra-colony exometabolome. The intra-colony fluid was shown to contain a plethora of metabolites, with the amino acids glutamine, glutamate and alanine being the most highly concentrated. Furthermore, histidine, leucine, methionine and uracil were all shown to be part of this exometabolome [49,50,51]. Interestingly, previous efforts to co-culture complementary auxotrophs had limited effectiveness in supporting co-growth in liquid cultures, indicating the importance of spatial structure in facilitating cooperation. The measurements were obtained from cells in the exponential growth phase, so here the effect was not influenced by cell aging. However, our recent data suggest that variability in the amino acid presence sensing in the exometabolome of starving colonies could influence the rate of transition to the quiescent state in yeast (Marek at al. 2020 in reviews). It seems that in suitable conditions, yeast easily establishes metabolite exchange, and that it may be a natural property of a growing yeast colony.

Overall, multiple researchers show that the presence of a structured environment and subsequent formation of colonies or other forms of yeast adhesion result in heterogeneity and cooperation within cells.

### 3.2. Starvation

Starvation is a well-known condition that triggers phenotypic heterogeneity within clonal yeast populations. As a response to carbon starvation, *S. cerevisiae* cells change the expression levels of metabolic genes [52,53], and actively differentiate into non-dividing forms. Diploid yeast can differentiate in multiple ways; they can sporulate to form haploid spores; they can switch into “pseudohyphal growth” (phg) to grow as elongated chains of cells and they can enter a stable nonproliferative state known as a “quiescent” (Q) state [54]. A fraction of cells remain non-differentiated, “non-quiescent” (NQ) [55]. Haploid yeast does not produce spores. In case of starvation, a fraction of the haploid yeast population differentiates into Q cells. Each of the cell types serves a different function within the yeast population.

The primary function of sporulation is to produce cells (haploid spores) that are more resistant to environmental stresses than the vegetative cells from which they derive. Sporulation is the most energy demanding of all the earlier- mentioned yeast forms [54]. In the presence of environmental structure, yeast colony sporulation patterns reflect differences in the nutrient environment across the community, as well as cell-to-cell signals within communities. Spores are found in the internal layer of cells and in a second layer of cells at the agar surface. The boundaries between sporulating and nonsporulating regions are very sharp, and dependent on the presence of nutrients [5].

One of the major functions of quiescence is the same as that of sporulation—resistance to environmental stresses. Like sporulation, quiescence is a response to nutrient deprivation that requires an energy investment [52,55]. The Q cells have thickened cell walls, more trehalose and other storage materials and denser vacuoles compared to growing and non-differentiated NQ cells [56]. All these features make them better adapted to persistence during a starvation period and help them restart divisions after a nutrient becomes available again. Glucose dependent yeast cells are also very sensitive to the signals of the glucose presence in the environment. The time at which individual cells resume growth after a period of starvation can vary greatly between genetically identical Q and NQ cells. This heterogeneity influences how quickly and efficiently populations react to an environmental oscillation in the available glucose level (M. Opalek, unpublished data). Q cells, after a lag phase, synchronously re-start mitotic divisions, while NQ cells are much more variable [57,58,59]. Interestingly, Q diploids are more resistant to environmental stress than growing cells, but less resistant than spores to environmental stress [60]. Q cells do not remain viable indefinitely. As time passes and Q cells age, their viability diminishes. Thus, quiescence, aging and eventual death can be considered progressive stages in a single differentiation pathway. Yeast cell starvation for different nutrients activates a quiescence-related transcriptional program that can involve various metabolic and genetic responses. Survival in carbon-starvation conditions requires functional mitochondrial and respiration genetic programs, whereas nitrogen starvation requires functioning vacuoles and autophagy genetic programs. The genetic programs necessary in phosphate starvation conditions overlap partially with those required during carbon and nitrogen starvation [52]. Altogether, these results show how starvation of essential nutrients or even less severe metabolic constraints can drive heterogeneity within the clonal cell community.

Not only the complete lack of glucose in the environment, but also glucose-limited growth conditions (0.1% instead of usually 2% rich laboratory medium) can cause metabolic diversification in initially isogenic yeast populations [61]. Within a mature, clonal yeast colony developing in low glucose conditions, cells arrange into metabolically heterogeneous, disparate cell groups that cooperate. A fraction of cells in such a community start to produce and provide a sugar called trehalose. Trehalose is a highly stable disaccharide of glucose. Trehalose, as an energy resource, can be used by other cells in such colonies that switch to use of the pentose phosphate pathway. Processing and gaining energy from trehalose is not the case while glucose is still available. These findings suggest that the availability of a specific, shared nutrient (in this case, trehalose), which can be made by the cells themselves, is a sufficient signal to trigger cells to specialize to a stress resistant phenotype.

### 3.3. Aging 

Baker′s yeast has a finite lifespan and ages in two ways: a mother cell can only divide a defined number of times, referred to as its replicative lifespan (RLS) [62]; and a non-dividing cell can only live a define period of time, referred to as its chronological lifespan (CLS) [63]. In constant environments, both types of cellular aging result in characteristic phenotypes and hence are an additional cause for heterogeneity [64,65,66]. Yeast ages with every division, and replicatively older cells show increases in size and changes in gene activation and metabolism [67]. Biosynthetic activities also change as cells’ age-proteins involved in translation become more abundant, relative to their transcripts, levels of pyruvate and TCA cycle intermediates and levels of amino acids decrease [68]. Moreover, features of starvation and oxidative stress are also induced at old ages [69]. Due to these changes, individual yeast cells also become more vulnerable to stress. Chronological aging also influences replication capacities—the longer cells are starved, the lower the population′s re-growth efficiency, even for replicative young cells [57,70].

*Sacharomyces cerevisiae* colonies also use volatile and diffusible compounds to communicate aging status with one another. In colony development in time, *S. cerevisiae* cells behave periodically with acidic and alkali phases [71]. When the cells switch from an acidic phase to an alkaline phase, they produce volatile ammonia as a pulse, which triggers ammonia production in surrounding colonies. The released ammonia signals for the synchronization of the nutrient starvation response. In contrast to individual cells, older colonies and mature biofilms are more resistant (as a group of clonal cells) to external toxins [72]. It is important to note that aging in colonies is coupled with the presence of structure in the environment. Thus, these factors are often difficult to assess independently.

## 4. Evolutionary Advantages of Yeast Multicellularity and Heterogeneity 

Forming heterogeneous, cooperating groups allows *S. cerevisiae* to perform tasks that cannot be as efficiently accomplished simultaneously by individual cells, or even groups of cells not evolved as communities. Such a strategy overall results in better adaptation to specific life conditions experienced by efficacy in nutrient utilization, the mechanism of sharing metabolites and coping with stress [42,51,73]. Nutritional stress is the most common one experienced by microorganisms. However, various methods of stress protection and stress tolerance are among the most commonly found adaptations in laboratory researched yeast communities. It is shown that the stress tolerance of a cell community does not necessarily reflect the stress tolerance of its individual cell members. Heterogeneity establishes substantial cell diversity in reaction to stress. Some cells continue to divide and grow, some become non dividing and others die upon application of the stressors. Below, we focus on examples illustrating evolutionary advantages of yeast multicellularity and heterogeneity.

Yeasts are adapted to use glucose as a main source of energy. The evolutionary advantage of multicellular populations in this main nutrient deficiency has been demonstrated. Polysaccharides like sucrose are not an easily accessible carbon source for yeast cells. However, *S. cerevisiae* cells secrete an enzyme, invertase, that breaks sucrose into fructose and glucose externally. Available as glucose, it can be easily used as a carbon source. It was shown experimentally that a single yeast cell, however, is unable to survive independently on such a low sucrose concentration, as most monosaccharides (glucose) diffuse away and become again inaccessible to the cell. In consequence, the cell is unable to intake enough resources from the environment to grow and divide. In multicellular clumps, however, all cells break down sucrose and the monosaccharide concentration in their microenvironment is higher, allowing the group to thrive successfully [14].

Yeast groups of adherent cells possess other clear fitness advantages—they generally form bigger biofilms than smooth colonies formed by planktonic cells. This advantageous effect is only possible when genetically identical *FLO11* cells heterogeneously express this flocculin. To realize this synchronized growth, Flo11^+^ cells form a kind of scaffold for the subsequent spread of clone-mate Flo11^-^ cells while generating the structured appearance of biofilm colonies. Such structure allows for more effective nutrient intake and, in consequence, a broader dispersal than the planktonic type of growth [42].

Transition into spores and quiescent cells in response to lack of glucose allows for longer survival and higher stress tolerance for the whole population [70,72]. *S. cerevisiae*’s glucose-sensing mechanism is very sensitive, resulting in a fast cell metabolic response to the glucose level in the environment. Stress resistance cells can survive long exposure to starvation and can be the source of colony repopulation [37]. Diversification into the quiescence and non-quiescence (proliferating) state of cells was shown to be adaptive also in environments with fluctuating access to glucose. A yeast population was sequentially subjected to two opposite environmental conditions—feast, when cells should divide as quickly and efficiently as possible, and famine, when transition to quiescence is crucial for survival. We found that a laboratory strain that presumably had been long adapted to a changing environment due to cyclic growth (serial transfer) on rich YPD-type medium, had higher fitness than either of the clones having a majority of one type of cell (NQ or Q) only [58].

Yeast stress response and metabolic cycles are two highly interdependent processes. Stress response involves metabolic re-configuration and metabolism determines not only the growth rate of cells, but also provides cofactors for the stress responsive machinery, and is a source of toxic or oxidizing molecules itself. As currently existing experimental approaches to study such metabolically driven quiescence/non-quiescence (proliferating) oscillations during feast/famine cycles are very limited, we need more evidence to support one of the existing evolutionary scenarios: (i) there could be production and secretion of a resource by a subpopulation of cells (“feeders”), which is taken up by other cells that will go on to divide; (ii) there could be secretion and accumulation of a metabolite that is sensed and taken up by only some cells and (iii) there is a build-up of a metabolite, which is consumed by the cells at some threshold concentration [61].

Biofilm colony structure protects inner cells from toxins, including antifungal drugs, via several adaptations. Mechanical protection is provided by the extracellular matrix and invasive growth characteristic for the bottom-forming cells. Multidrug resistance transporters localized in the areal surface of the colony allow for increased resistance to multiple toxins. All this results in the fact that the biofilm′s resistance to toxins is a major challenge for curing infection [37,73]. 

Cooperation between cells in multicellular clusters is crucial in order to establish metabolic dependencies. Evolution of a cluster′s reproduction is an example of division of labour. To allow further division, a big cluster needs to break into smaller ones—some cells inside that cluster must undergo programmed cell death to allow separation. Fitness consequences were studied on snowflake yeast, which reproduces by breaking big clusters into smaller ones [17]. Apoptosis was shown to be a trait co-evolving with increased cluster size, not a direct effect of its size. As such, apoptosis is interpreted as an adaptive altruistic trait underpinning cellular division of labour [74].

## 5. Conclusions and Future Directions

Collections of ecologically diverse strains could be used to define rules and identify regulatory pathways that are common to different structures and may form the basis of yeast multicellularity [37,75]. For now, it is mainly laboratory *S.cerevisiae* strains that are being explored. However, there are new resources to research world yeast collections that are often also sequenced, as already described in detail. It is important, however, to remember that in the case of *Saccharomyces* spp., multicellularity is a reversion, not a new novel growth form [3]. What we learn about selection for multicellularity in yeast has to be taken in this context of a secondarily single-celled organism. Using collections of ecologically diverse stains allows researchers to look for changes in the genome caused by natural selection, which is much more multidimensional and usually weaker than artificial selection applied in laboratories.

The Microbiology field is expanding rapidly in all dimensions. We see the use of contemporary advancement technologies that allow research to be scaled up to interspecies population interactions, scaled down to the microenvironment of a single cell and moved from laboratories to natural ecological conditions. This allows us to build up the knowledge regarding drivers of heterogeneity [36]. All these promise quick progress in this field in the near future.

## Glossary

Stay together strategy: One of the possible types of multicellular group formation, where cells reproducing vegetatively by budding form groups by cell-cell adhesion or incomplete cell wall separation, where the daughter cell stays attached to its mother cell.
Come together strategy: One of the possible types of multicellular group formation, where individual cells adhere to each other by cell wall proteins.
Colony, non-adhesive colony: Structures formed on solid substrates exposed to air. Cells that do not produce adhesins are sessile and will develop non-adhesive colonies. This is the typical growth form of many laboratory strains on solid agar media [11].
Biofilm: Multicellular structure (adhesive colony) on a semisolid medium. Typically, cells in biofilms adhere to each other and to the foreign surface [11].
Mat: Complex multicellular biofilms, which are formed on low density (0.3%) YPD agar, which are able to float on the liquid surface [11,37].
Stalks: Stalk structures growing vertically from small holes in the agar. *Saccharomyces cerevisiae* stalks are 5–30-mm long differentiated structures composed of two distinct layers, identified by ultrastructural analysis, of stalk thin sections. The central core, which is composed of yeast cells and spores, is surrounded by surface layers of highly vacuolized cells with thick cell walls. These cells are dying (or already dead) and seem to protect the structure against drying and other environmental effects, forming a skin-like structure [37].
Flocs: Clumps of cells created by cell-to-cell adhesion that may sediment to the bottom. Cells can stick to each other due to cell wall proteins—flocculins [11].
Flors: Specific types of interfacial biofilm on air–liquid self-adhesion groups that can float on the liquid surface [11].
Division of labour: Three conditions are needed to identify a division of labour: (a) phenotypic variation, reflecting different tasks undertaken by individuals within a population; (b) cooperation between individuals performing different tasks and (c) adaptation—that the behaviour maximizes the inclusive fitness of all individuals involved [33].
Public goods: Resources produced by cooperating organisms, which are available in the environment and can be used by other organisms in the population.
Cooperation: Interaction in which organisms perform a costly task together for the common benefit (for example, production of a public good), which can be used by all population members.
Homologous recombination: Method of genetic modification in which chromosomal genes are swapped with genes on plasmid (which can be designed by the researcher), so that a gene of interest is transferred to the chromosome.
